# Robotic-Assisted Minimally Invasive Direct Coronary Artery Bypass Grafting: A Surgical Technique

**DOI:** 10.3390/jcm13082435

**Published:** 2024-04-22

**Authors:** Laura Giroletti, Ascanio Graniero, Alfonso Agnino

**Affiliations:** Department of Cardiovascular Surgery, Division of Robotic and Minimally-Invasive Cardiac Surgery, Humanitas Gavazzeni-Castelli, 24125 Bergamo, Italy; ascanio.graniero@gavazzeni.it (A.G.); alfonso.agnino@gavazzeni.it (A.A.)

**Keywords:** robotic-assisted cardiac surgery, minimally invasive coronary surgery, robotic-assisted mammary artery harvesting

## Abstract

In recent years, there has been a growing interest in robotic-assisted coronary artery revascularization in Europe. Two different types of surgery can be performed using a robotic platform: RA-MIDCAB, in which the mammary artery is harvested endoscopically with robotic assistance and off-pump bypass graft is achieved under direct vision through mini thoracotomy, and TE-CAB, completely robotically performed. We started the robotic cardiac surgery program for mitral valve disease in our hospital, Humanitas Gavazzeni (Bergamo, Italy), in 2019; and in 2021, we addressed our experience with RA-MIDCAB. After a learning curve period, we have developed our technique to optimize the benefits offered by the robotic platform, tailoring strategy to individual patients, based on preoperative radiological images.

## 1. Introduction

In the heterogeneous scenario of minimally invasive coronary surgery, the birth of the modern minimally invasive direct coronary artery bypass grafting (MIDCAB) procedure occurred in 1994 thanks to Bennetti and Ballester, who reintroduced the left mini-thoracotomy approach for LAD revascularization in combination with a thoracoscopic left interior mammary artery (LIMA) harvest [1].

As a further advancement, the first robotic-assisted coronary artery revascularization was described in 1999 by Carpentier and Loulmet in Paris [2], but only in recent years there has been growing interest in this technique in Europe, where the number of centers adopting the procedure doubled between 2016 and 2019 [3].

Improvements in robotic technology have played a key role in this change, and two different types of coronary revascularization surgery can currently be performed using a robotic platform: RA-MIDCAB, in which the mammary artery is harvested endoscopically with robotic assistance and off-pump bypass graft is achieved under direct vision through mini-thoracotomy; and TE-CAB, which is completely robotically performed. Several studies have demonstrated the safety and efficacy of the procedure in terms of post-operative complications and graft patency [4,5,6].

We started the robotic cardiac surgery program in our hospital in 2019, initially focused on treating mitral valve disease. In 2021, we expanded our experience to include coronary surgery as a treatment for single- or multivessel disease in the context of hybrid revascularization.

In this paper, we will describe the RA-MIDCAB surgical technique adopted in our center, focusing on technical details of both preoperative patient management and the operative phase.

## 2. Surgical Technique

### 2.1. Preoperative Diagnostics Work Up

**Figure 1:**
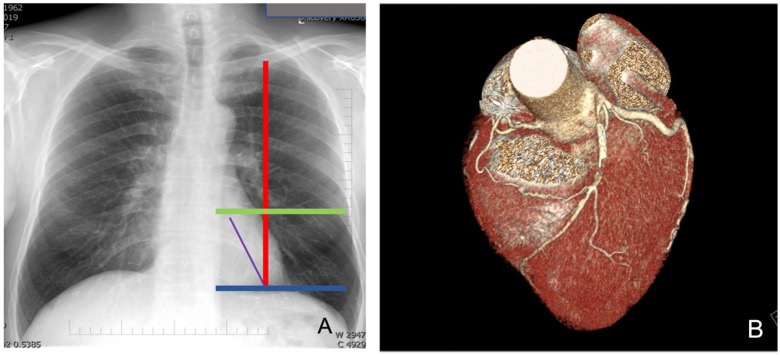
Preoperative chest X-ray (**A**) and CT-scan with 3D reconstruction (**B**).

Computed tomography (CT) (third-generation 192-slice dual-source SOMATOM Force, Siemens Healthineers, Erlangen, Germany) and chest X-ray are commonly performed to delineate the heart anatomy and its relationships with the surrounding thoracic structures (Figure 1A,B).

More specifically, chest X-ray provides an initial indication of the left heart ventricle volume and the position of its free margin relative to the chest wall and intercostal space. As depicted in Figure 1A, we mark the mid-clavicular line and two horizontal lines that include the left second cardiac arch, corresponding to the left ventricle, allowing us to identify the possible position of the LAD with respect to these markers. This is very important to guide the positioning of ports, particularly the port for the endoscopic camera and for anchoring the left robotic arm; a bigger left ventricle requires a more medial port location to avoid competition between the instruments and beating heart. In addition, the LAD position can give indications on the effectively lateral site of the left mini-thoracotomy required for bypass grafting after mammary artery harvesting.

A CT scan or CT-ANGIO scan is useful in preoperative planning because it gives data on the size, position and quality of the left internal mammary artery and left anterior descending coronary artery (LAD). Indeed, the degree and site of any calcification of target vessels and any intramyocardial course of LAD can be assessed. A CT scan with 3D reconstruction can more accurately describe the relationship between the LAD and the chest wall, aiding in the selection of the intercostal space for thoracotomy.

Finally, radiological cardiac imaging provides information about the chest wall anatomy, facilitating port placement.

Pre-operative management is completed with spirometry, testing of the diffusing capacity of the lungs for carbon monoxide (DLCO), transthoracic echocardiography, electrocardiography and routine biochemical assessment. The pulmonary function test is important for the anesthesiologist to measure the lungs’ function and ability to transfer gas from inspired air to the bloodstream. In this way, it is possible to predict the patient’s response to mono-pulmonary ventilation, which is required for the robotic procedure. Severe pulmonary disease could contraindicate RA-MIDCAB.

In Table 1, we summarize key parameters of preoperative work up.

### 2.2. Surgical Technique

In routine general anesthesia, a double-lumen endotracheal tube is placed for selective lung ventilation. Alternatively, the left lung is excluded with an endoluminal balloon.

To start the procedure, the patient is positioned in a supine posture with a square pad strategically located behind the left scapula to moderately bump up the left hemithorax.

**Figure 2:**
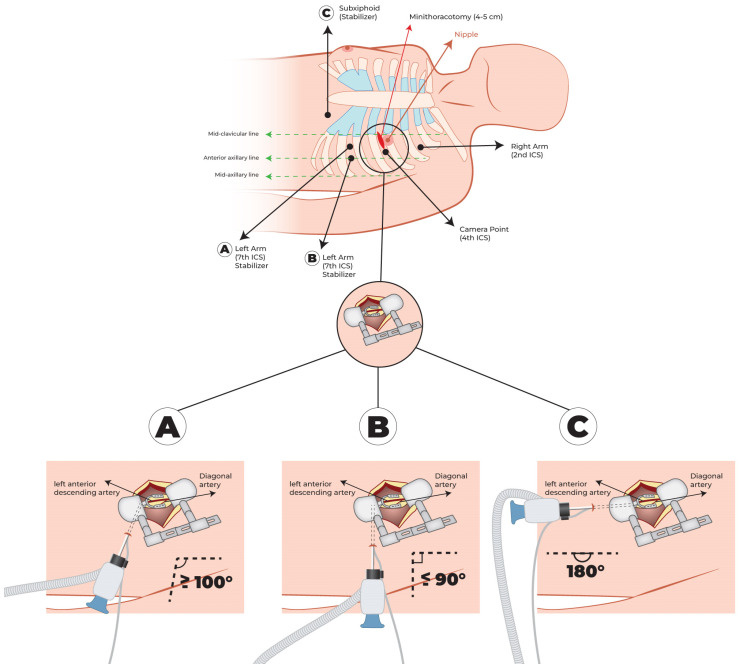
Second, fourth and seventh, intercostal space marked to guide ports positioning. Fourth or fifth intercostal space defined for mini-thoracotomy. (**A**–**C**) possible sites of coronary stabilizer according to patient’s chest and heart anatomy and resulting angle between the arm and the terminal movable portion of the stabilizer.

As depicted in Figure 2, the surgeon marks the second, fourth and seventh left intercostal space to guide the positioning of ports. Based on radiological images, they also define the fourth or fifth intercostal space for mini-thoracotomy.

Initially, both lungs are excluded from ventilation, and CO_2_ is insufflated with a needle into the left hemithorax at a mean pressure of 4–10 mmHg to collapse the left lung, prevent mediastinal shift related to right lung hyper expansion and move the heart to the right to create space. Then, only the left lung remains excluded. The target CO_2_ pressure is influenced by the size of the heart: a small heart allows for the creation of space with lower pressure levels.

The first 8 mm port, the site of the endoscopic camera, is placed in the fourth intercostal space, between the nipple and the anterior axillary line, especially in the dilated heart. Under endoscopic vision, a second 8 mm port is positioned in the second intercostal space, between the midclavicular and anterior axillary line. Some surgeons insert the third 8 mm port in the seventh intercostal space at the level of the anterior axillary line: we prefer to place this port in the seventh intercostal space medially to the anterior axillary line, as shown in Figure 2A. We chose this site because later, when the LITA harvest is completed, the arm of the coronary stabilizer (Medtronic, Minneapolis, MN, USA, Octopus Nuvo Tissue stabilizer) is placed here, and we note a better angle, greater or equal to 100°, between the arm and the terminal movable portion of the stabilizer, simplifying placement on the left ventricular wall. This position ensures good stability of Octopus Nuvo at the anastomotic site while, with this angle, its rigid arm remains further from the apex and wall of the left ventricle, reducing the possibility of conflict and pressure on the heart and improving hemodynamic stability during bypass. It is also important to check the distance from the iliac spine to avoid conflict with the external support of the stabilizer arm.

A more lateral position of the third port, described in Figure 2B, provides an angle less or equal to 90°; in this case, we note, in the reduced space of the mini-thoracotomy, less comfort in the positioning of the stabilizer on the cardiac surface and more difficult LAD stabilization. We also observe increased interference between the arm of the stabilizer and left ventricle, with greater influence on hemodynamic parameters.

Another strategy is to create a distinct subxiphoid access to place the stabilizer arm (Figure 2C). This approach allows for the best angle between the arm and the terminal movable portion of the stabilizer (near 180°), almost parallel to the position of the LAD, enabling the best stability at the anastomotic site and minimal interference with patient hemodynamics. However, in this case, a surgical site is added with major surgical trauma, and sectioning of the xiphoid is required.

Therefore, customizing the port placement strategy based on radiological imaging is crucial for optimizing the technique.

Carbon dioxide (CO_2_) insufflation is continued through the third port with a target intrathoracic pressure of 6–10 mmHg. The Da Vinci X robot system (Intuitive surgical, Inc., Sunnyvale, CA, USA) is placed at the patient’s right side, and robotic arms are anchored to the designated working ports. A 30° endoscopic camera (Intuitive) is inserted in the second port to navigate the thorax, pericardium, and heart structures with enhanced views (10× magnification).

The surgeon carefully opens the pericardium above the phrenic nerve; pericardial fat is removed if necessary. A second hole in pericardium is realized under the phrenic nerve; in case of bleeding or pericardial effusion, this hole drains the fluids in the left pleura.

**Figure 3:**
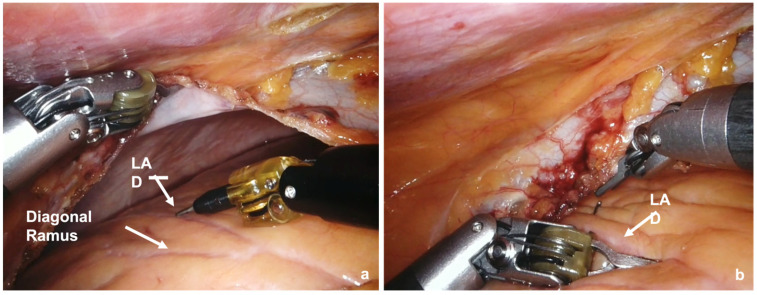
(**a**,**b**) LAD and an anastomosis site identification.

The LAD and a possible anastomosis site are identified, and a clip is placed near the point to guide the surgeon (Figure 3).

The full length of the LITA graft is harvested using a low-energy monopolar electrocautery spatula and high-energy bipolar cautery forceps. The mammary artery is collected according to a skeletonized technique: the surgeon first opens the thoracic fascia surrounding the LITA and gently dissects the connective tissues between it and the medial mammary vein, then moving on to the lateral mammary vein. Bipolar cautery forceps are used to seal and divide small collateral branches, while metal clips are used for larger size branches.

**Figure 4:**
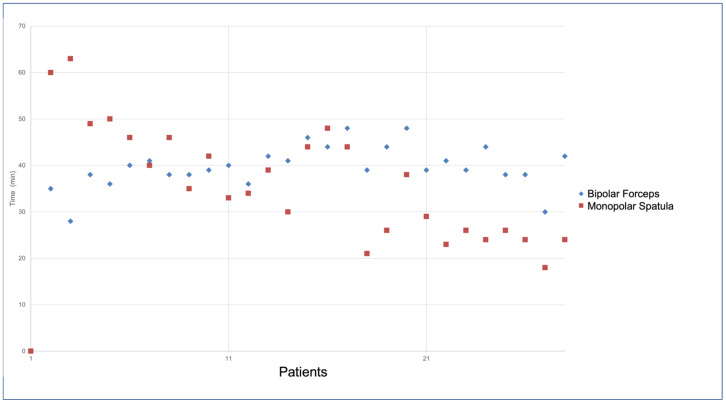
The shift in instrument use during the “learning curve” in RA LITA harvesting. Red dots identify the monopolar spatula; blue dots identify the bipolar forceps.

As depicted in Figure 4, there was a shift in instrument use during the “learning curve”. In the first eleven patients, a monopolar spatula was preferred for tissue dissection and LITA harvesting; subsequently, we more frequently used bipolar forceps indiscriminately on the left or right robotic arms.

The time of instrument use was defined by consulting the My-Intuitive App and extrapolating the times from the records of individual cases.

When the harvest is completed, LITA is closed with a bulldog and divided distally after administration of heparin: the remaining stump is sealed with metal clips.

**Figure 5:**
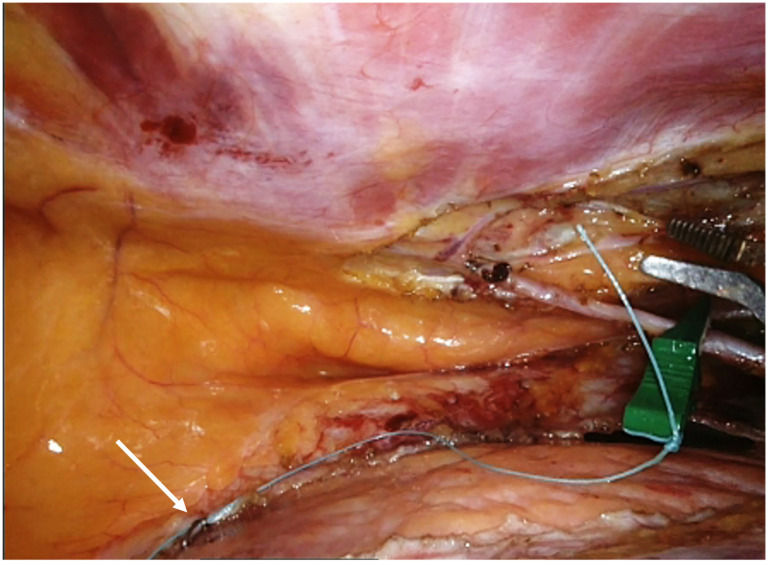
To simplify mammary recovery during the next stage of coronary anastomosis, a silk thread is tied to the bulldog and attached to the pericardium.

To simplify mammary recovery during the next stage of coronary anastomosis, we tie a silk thread to the bulldog and then attach it to the pericardium (Figure 5).

Based on the LAD site previously identified with a clip, an appropriate intercostal space is determined using a needle inserted through the chest wall, and mini-thoracotomy (4–5 cm) is generally performed at the fourth or fifth intercostal space.

A suction, a flexible stabilizer (Medtronic, Minneapolis, MN, USA, Octopus Nuvo Tissue stabilizer), is placed to reduce cardiac motion at the site of anastomosis using vacuum. The stabilizer is composed of a stiff arm with a removable blunt tip, a detachable stabilizer headlink with a ball joint that provides increased flexibility and range of motion, and a vacuum line extension. Initially, we position the head of the stabilizer on the heart, and through the site of third port (after its removal), we tunnel the vacuum line outside the thoracotomy. We insert the arm with the blunt tip through the same previous seat of the third port and, after removing the tip, we anchor the arm to the stabilizer head, activating the vacuum at −400 mmHg. The arm is fixed to an external support.

**Figure 6:**
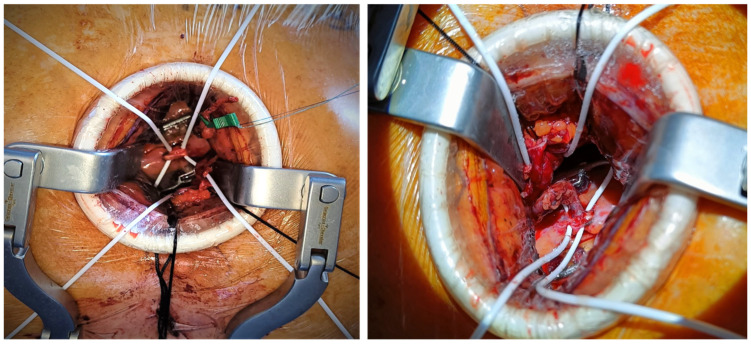
Off-pump anastomosis of LITA to LAD.

The off-pump anastomosis of LITA to LAD is realized under direct vision according to the surgeon’s standard technique. In our experience, we isolate and expose LAD, surrounding it with bioloop silicon string (Biomed, Ragusa, Italy), and use a coronary shunt (Figure 6). Finally, intraoperative graft quality control is performed using a Transit Time Flowmeter (Mira-Q, Medistim, Plymouth, MN, USA), which, by establishing numerical parameters such as the pulsatility index (PI), diastolic filling (DF%), and mean flow, provides an accurate view of the dynamics of graft function.

In our experience, a *fast-track* protocol including rapid extubation, mobilization and reduction in ICU length of stay should be applied if the patient has specific characteristics, i.e., no bleeding, no inotropic drugs and hemodynamic stability, correct blood gas values, biochemical profile of blood tests in the normal range, and no neurological deficits.

We have observed a higher percentage of patients who are extubated directly in the operating room, resulting in early mobilization in the ICU a couple of hours after surgery. By minimizing the size of the incisions, the operative trauma for patients is limited: local infiltration with anesthetic drugs at the level of surgical sites further reduces postoperative pain. If the clinical status is stable and there is no bleeding, invasive devices such as a chest drain and bladder catheter are removed within 24 h, upon which the patient can leave the intensive care unit. The hospital length of stay is approximately five to seven days, wherein patients follow a physiotherapy program of cardiopulmonary rehabilitation. Finally, if no major complications occur, most patients are discharged home.

Clinical follow-up includes the visit and electrocardiographic and biochemical monitoring at about two months after surgery, then at six months and then annually.

TT-echocardiographic examination is scheduled at six months after surgery, while second-level examinations (stress TT Echo, coronary CT scan, etc.) are scheduled in case of the appearance of symptoms or alteration in symptoms from first-level examinations.

Patients in a hybrid revascularization protocol will complete the coronary angioplasty procedure with a timeline that depends on the characteristics of the coronary artery disease.

### 2.3. Pitfalls in RA-MIDCAB

While obesity, elderly, REDO operation or chronic pulmonary diseases were previously considered to be contraindications for robotic coronary surgery, the robotic population has now expanded, and there is no absolute contraindication even in “high risk” patients.

However, some pitfalls must be considered in RA-MIDCAB, particularly during preoperative evaluation.

Incorrect assessment of the anatomy of the chest wall and heart and the relationships between the two structures can result in errors in port placement. Radiological imaging is essential for planning surgical strategy. For example, in patients with a dilated left ventricle, a more lateral position of the ports may result in contact and conflict between the instruments and the beating heart, with instability of the instruments and possible influence on hemodynamic parameters. In addition, robotic LITA harvesting in this case present greater difficulty and a higher risk of injury to the artery.

Special care must be taken when choosing the site of the third port, where, in the second step, the coronary stabilizer arm will be placed. As previously described, a correct angle between the arm and the terminal movable portion of the stabilizer is important to ensure the greatest comfort while performing the anastomosis, and an error can make surgical action more difficult.

It is also important to consider the possible conflict between the robotic arms and the patient’s clavicle or left iliac spine.

If you feel that the port placement or intercostal space of the mini thoracotomy is not optimal, do not hesitate to modify the site to work as comfortably as possible.

Due to incorrect coronary CT scan evaluation, the intramural course of VATS may not be identified, with a subsequent need for conversion to sternotomy.

A second point concerns the robotic technology and the significance of the learning curve to acquire confidence with robotic tools. We note that, at the beginning of the experience, the absence of the tactile reflex on robotic instruments may result in damage to the mammary artery during harvesting, so it is necessary to work in a calm environment and with longer times than standard surgery, if necessary.

In the first step, RA-MIDCAB requires the use of CO_2_ to create a sealing system with an intrathoracic pressure of 6–10 mmHg and mono-lateral ventilation with left lung exclusion; a possible complication in this case is hemodynamic or respiratory instability with patient desaturation and hypotension. For this reason, the role of the anesthesiologist is crucial to the success of the procedure.

Finally, postoperative hemostasis not only at the site of the anastomosis but also at the port site and LITA bed is critical to avoid revisions for bleeding and transfusions. During LITA harvesting, CO_2_ insufflation controls any venous micro-bleeding, but after mini-thoracotomy is performed and intrathoracic pressure is reduced, bleeding may occur at any site, and a final check is important before closing the surgical sites.

## 3. Discussion

If minimally invasive direct coronary artery bypass graft (MIDCAB) performed with an off-pump technique via small left anterior mini-thoracotomy is established to be safe and effective with excellent short- and long-term results [7,8,9], the use of robotic assistance during LITA harvest may further benefit the surgeon, improving their comfort and the quality of the surgical procedure.

The 2018 ESC/EACTS Guidelines on myocardial revascularization [10] recommend considering minimally invasive CABG through limited thoracic access in patients with isolated LAD lesions or in the context of hybrid revascularization (class IIa B) in centers with expertise. MIDCAB is not distinguished in the text from RA-MIDCAB in terms of clinical indications.

However, in several hospitals with experience in MIDCAB, the introduction of robotic technology results in a change, with more patients treated using RA-MIDCAB.

In gaining experience in robotic surgery, the physical characteristics of patients are not considered as selection criteria, allowing for “high risk” patients to be treated with this technique.

In a recent paper, Gofus and colleagues compared conventional MIDCAB and RA-MIDCAB, demonstrating, at equal outcomes in terms of mortality and perioperative complications, the advantages associated with robotic platform use [11].

In MIDCAB, a short left anterior thoracotomy is made at the fourth intercostal space and LITA is harvested completely under direct vision with the help of specialized retractor.

In RA-MIDCAB, high-definition exposure, three-dimensional visualization and excellent precision enabled by robotic instruments allow mammary artery harvesting with greater comfort and control with respect to conventional techniques, further minimizing surgical access and avoiding rib spreading, dislocation and fractures.

Robotic technology facilitates access to proximal and distal parts of the graft, increasing its length with respect to MIDCAB, while detailed visualization of LITA secures the side branches with better precision and provides better bleeding control from the LITA bed after taking the vessel down.

In addition to clinical and surgical benefits, RA-MIDCAB does not compromise the excellent graft patency, as reported in several studies [12,13].

We introduced RA-MIDCAB into our armamentarium in 2021, about 3 years after the first robotic cardiac surgery performed on the mitral valve.

The learning curve associated with robotic-assisted mitral valve surgery (RA-MVS) was complete not only for the surgeon but also for the entire team [14]; for this reason, we expected an automatic transition to RA-MIDCAB, but that did not happen.

Differently from RA-MVS, robotic-assisted mammary artery harvesting is performed with a beating heart without the use of a cardiopulmonary bypass; thus, the range of motion and maneuverability of the robotic arms in the chest must be taken into consideration to avoid damage to the cardiac structures.

While in mitral surgery the thoracic cavity and the outside are in communication through a working port, in RA-MIDCAB, the surgeon initially works in a sealing system, where carbon dioxide (CO_2_) insufflation is continued to achieve an intrathoracic pressure of 6–10 mmHg. This aspect ensures better vision and control of any venous micro-bleeding but also requires careful anesthesiologist management to maintain hemodynamic stability and proper metabolic balance. The team is still crucial to ensure the success of the procedure and achieve excellent patient outcomes.

Another point concerns the left-side approach, an uncommon element for the cardiac surgeon in mammary artery harvesting, which requires a change in mindset and technique. Furthermore, on the left thoracic side, the robotic arm encounters greater potential conflict with the left shoulder and the iliac spine. For these reasons, proper pre-operative management of patients with radiological imaging plays a key role in RA-MIDCAB, and the experience gained with RA-MVS has been critical. Examination of Rx chest and CT scans, especially with 3D reconstruction, allows the position of working ports and the site of mini-thoracotomy to be modified according to the anatomical characteristics of patients.

We note that, by positioning the port in the seventh intercostal space more medially than the anterior axillary line, we have greater comfort in LITA harvesting, although it is necessary to pay attention to the competition with the left iliac spine. We can also use this site to place the coronary stabilizer arm, improving the angle between the arm and the movable end part of the stabilizer (≥100°) and simplifying its positioning on the heart. In this way, a new subxiphoid access is not needed for this instrument, reducing surgical trauma.

However, in other patients, subxiphoid access is preferred for the optimization of the technique in terms of greater comfort in performing a bypass because the Octopus Nuvo has a position almost parallel to LAD as well as better hemodynamic stability.

These considerations still demonstrate the importance of tailoring the technique to the patient to maximize the benefits of robotic support itself.

In RA-MIDCAB, bipolar cautery forceps are introduced as a new tool in our setting compared with RA-MVS. We note that, in the initial stage, the use of the bipolar cautery forceps during LITA harvest was low (about 30%), while we preferred a “more confident and safer” instrument: the monopolar electrocautery spatula. Over time, the ratio reversed with greater use of bipolar cautery forceps; this shift reflected growing confidence in both the tools and techniques employed and also influenced the harvest time.

In conclusion, a new “learning curve” specific to RA-MIDCAB is needed, although prior experience in robotic surgery provides an advantage in mastering robotic technology.

A careful study of the patient in the pre-operative period is essential for better planning the surgery and maximally exploiting the advantages of the robotic platform. Most of the mistakes and complications result from incorrect preoperative evaluation, as previously described.

Knowledge of robotic instruments is progressively enabling the study of technical expedients that can improve the comfort and performance of the surgeon, enabling the optimization of the procedure time and results.

## 4. Conclusions

The use of robotic technology in coronary artery surgery can be considered a valuable resource in the surgical armamentarium for the treatment of patients. The learning curve is essential, even if there is already experience in robotic surgery, for creating an ideal setting that is comfortable for the surgeons and allows them to optimize the benefits offered by the robotic platform. Clinical studies are needed to demonstrate the benefits of RA-MIDCAB; however, based on our experience, we have positively explored its potential.

## Figures and Tables

**Table 1:** Key parameters of preoperative work up (TT: transthoracic; DLCO: diffusing capacity of the lungs for carbon monoxide).

	Objective
**Chest X-ray**	Indication about the left heart ventricle volume and the position of its free margin relative to chest wall and intercostal space, to guide port positioning and site of the left mini thoracotomy
**CT Scan (optional 3D reconstruction)**	Size, position, and quality of left internal mammary artery and left anterior descending coronary artery (LAD). The degree and site of any calcification of target vessels and any intramyocardial course of LAD can be assessed.
**TT-Echocardiography**	Assessment of left ventricle function and analysis of segmental wall motion; exclusion of valvular disease
**Spirometry with DLCO test**	Assessment of the lungs’ function and ability to transfer gas from inspired air to the blood-stream, in order to predict the patient’s behavior toward the mono-pulmonary ventilation, required for the robotic procedure

## Data Availability

No new data were created.
